# Unidirectional Alignment of Surface-Grafted ZnO Nanorods in Micrometer-Thick Cells Using Low-Molecular-Weight Liquid Crystals

**DOI:** 10.3390/molecules27030689

**Published:** 2022-01-21

**Authors:** Kaho Ogata, Kohsuke Matsumoto, Yoshiaki Kobayashi, Shoichi Kubo, Atsushi Shishido

**Affiliations:** 1 Laboratory for Chemistry and Life Science, Institute of Innovative Research, Tokyo Institute of Technology, R1-12, 4259 Nagatsuta, Midori-ku, Yokohama 226-8503, Japan; kogata-st@polymer.res.titech.ac.jp (K.O.); kmatsumoto-st@polymer.res.titech.ac.jp (K.M.); ykobayashi-st@polymer.res.titech.ac.jp (Y.K.); 2 Department of Chemical Science and Engineering, School of Materials and Chemical Technology, Tokyo Institute of Technology, 2-12-1 Ookayama, Meguro-ku, Tokyo 152-8552, Japan

**Keywords:** nanorod, alignment, liquid crystal, surface modification

## Abstract

Inorganic nanomaterials such as nanotubes and nanorods have attracted great attention due to their anisotropic properties. Although the alignment control of inorganic nanomaterials is key to the development of functional devices utilizing their fascinating properties, there is still difficulty in achieving uniform alignment over a large area with a micrometer thickness. To overcome this problem, we focused on liquid crystals (LCs) to promote the alignment of anisotropic nanomaterials, taking advantage of the cooperative motion of LCs. We present the uniform, one-dimensional alignment of ZnO nanorods along the direction of LCs in micrometer-thick cells by grafting nematic LC polymers from the nanorod surfaces to provide miscibility with the host LCs. Polarized optical microscopy and polarized UV–visible absorption spectroscopy revealed the unidirectional alignment of nematic LC polymer-grafted ZnO nanorods parallel to the nematic host LCs.

## 1. Introduction

Inorganic anisotropic nanomaterials such as nanotubes and nanorods have attracted great attention due to their remarkable properties derived from their size effects and anisotropic shapes, such as electrical conductivity [1,2], optical properties of polarized emission and absorption [3,4], and thermal conductivity [5,6]. Various devices have been proposed as promising applications for nanomaterials—for example, piezoelectric energy harvesting [7,8], photovoltaics [9,10,11], and photonics [12]. To fully utilize their fascinating properties in functional devices, it is essential to control the alignment of nanomaterials at device-relevant length scales. To date, various alignment methods have been reported. For example, vertically aligned nanorods on substrates have been fabricated by metal organic chemical vapor deposition [13,14], pulsed laser deposition [15], hydrothermal growth [16], chemical bath deposition [17], surfactant-assisted sol–gel processes [18], and dopant mediated assembly [19]. All these methods are applicable to substrates satisfying the condition of the controlled growth of materials. Alternatively, methods for aligning presynthesized nanorods could expand the versatility of nanorod materials. Electric/magnetic fields [20,21,22], mechanical forces [23], and selective incorporation into microphase-separated block copolymers [24,25,26] have been used to induce the alignment of nanorods. However, there still remain challenges in unidirectional alignment over a large area, mostly due to aggregation.

Liquid crystals (LCs), which have both fluidity and anisotropy, are candidate materials for promoting nanorod alignment. Nanorods surface-modified with thermotropic LC molecules have been proposed for the utilization of anisotropic properties with regulated structures [27,28,29,30]. The formation of lyotropic LCs from nanorods modified with block copolymers has also been shown to utilize LC properties [31,32,33]. These studies suggest that the control of interactions between nanorods and LCs is key to their alignment. In order to further enhance the interaction between nanorods and LCs and effectively induce uniform alignment, we carefully regulated the surface density of LC polymers modified in inorganic nanorods and achieved the unidirectional alignment of zinc oxide (ZnO) nanorods over a large area [34]. Our concept of the surface-density regulation of LCs in nanorods allows cooperative molecular interactions with the surrounding low-molecular-weight host LC molecules, leading to hierarchical, uniform alignment without the aggregation of nanorods. However, the control of the alignment in the thickness direction is limited to only a few tens of nanometers near to the alignment layer. If the alignment of nanorods can be controlled to the thickness of micrometers, their applications as functional anisotropic materials will be greatly expanded by utilizing the nanoscale anisotropic properties on a macroscopic scale.

In this study, we report the alignment behavior of the LC polymer-grafted ZnO nanorods in an LC cell with a thickness of approximately 5 µm. Low-molecular-weight LCs with a high fluidity and the ability of molecular alignment are adopted as host LCs to drive the nanorods surface-grafted with LC polymers. We evaluate the miscibility of the surface-modified nanorods to the host LCs, which is the key to inducing the cooperative motion. Finally, we investigate the optical properties of the surface-modified nanorods in the host LCs in 5 µm-thick cells and demonstrate their unidirectional alignment in homogeneous and homeotropic manners.

## 2. Results and Discussion

### 2.1. Liquid Crystalline Behavior of the Nematic LC Host Dispersed with LC Polymer-Grafted Nanorods

**Figure 1:**
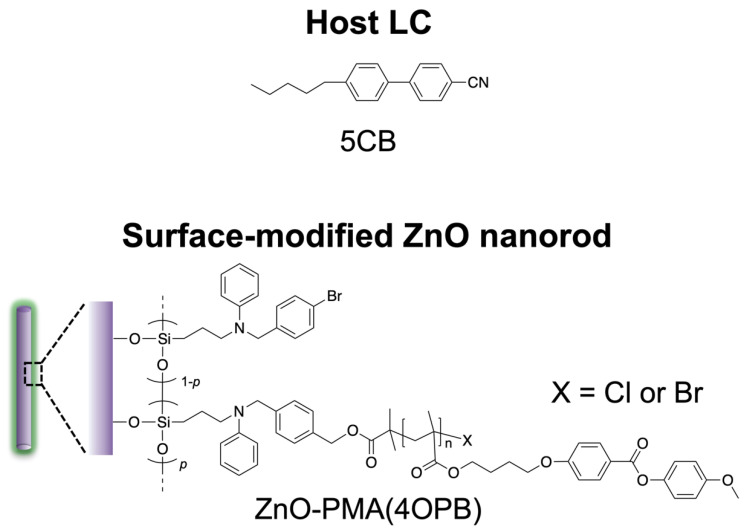
Chemical structures used in this study.

**Figure 2:**
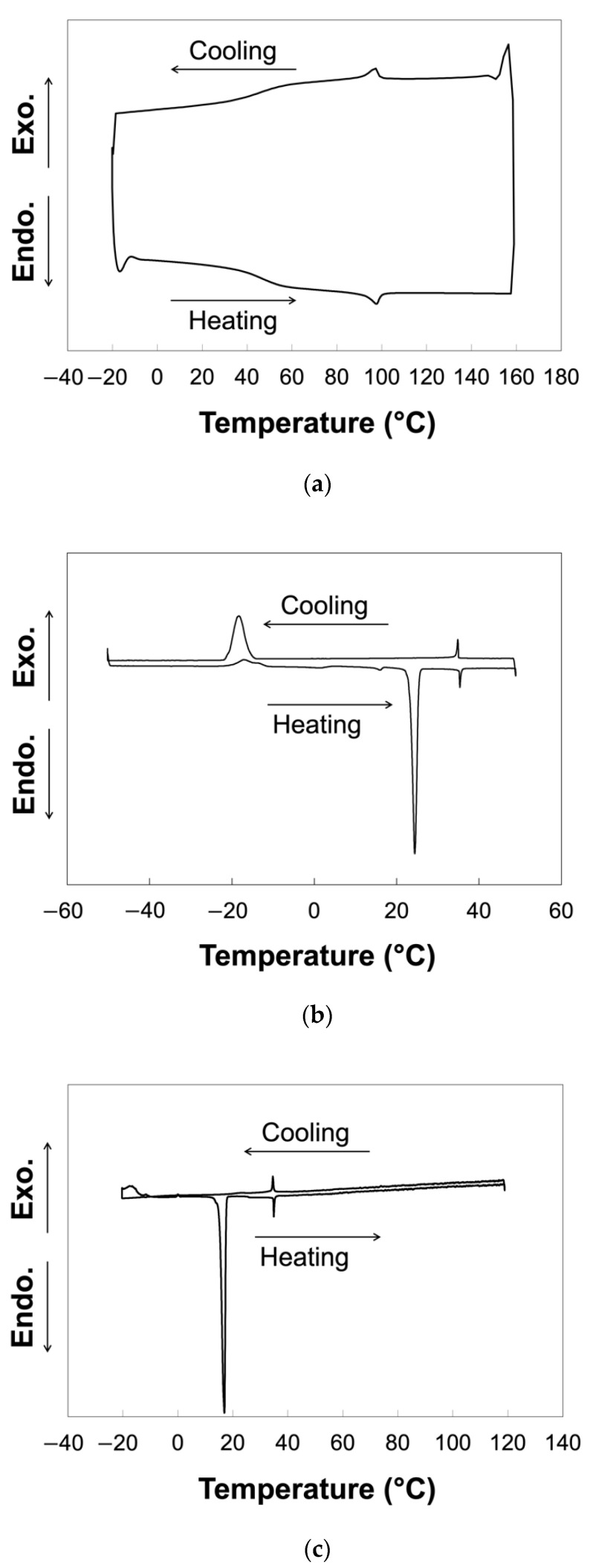
Differential scanning calorimetry (DSC) thermograms at 3rd heating and cooling cycle: (**a**) PMA(4OPB)-grafted ZnO nanorods; (**b**) 5CB; (**c**) their mixture. Scanning rate: 10 °C/min (**a**) and 1 °C/min (**b**,**c**).

We anticipated the alignment of nanorods by the cooperative interaction of host LCs and nematic LC polymer grafted from nanorod surfaces. Here, we adopted 4-cyano-4′-pentylbiphenyl, 5CB, as a low-molecular-weight host LC to induce the alignment of ZnO nanorods grafted with the nematic LC polymer, poly{4-[4-(4-methoxyphenyloxycarbonyl)phenoxy]butyl methacrylate}, PMA(4OPB), as shown in Figure 1. Firstly, the LC behavior of PMA(4OPB)-grafted ZnO nanorods dispersed in 5CB was investigated by differential scanning calorimetry (DSC) to evaluate their miscibility. Figure 2 shows the DSC thermograms of PMA(4OPB)-grafted nanorods, 5CB, and their 1:20 mixture in weight ratio. PMA(4OPB)-grafted nanorods showed an endothermic peak at 98 °C during the heating process and an exothermic peak at 97 °C during the cooling process (Figure 2a). Compared with the previous report [34], these peaks were attributable to the nematic to isotropic phase transition of the grafted PMA(4OPB). The nematic to isotropic phase transition enthalpies (Δ*H*_N-I_), expressed as total energy per molar quantity of LC mesogens, were 0.59 and 0.56 kJ/mol for the heating and cooling processes, respectively. 5CB showed a broad exothermic peak at around −15 °C derived from recrystallization, endothermic peaks corresponding to the melting at 24 °C, and the nematic to isotropic phase transition at 35 °C during the heating process, while the exothermic peaks of the isotropic to nematic phase transition were at 35 °C and the crystallization was at –18 °C during the cooling process (Figure 2b). Both Δ*H*_N-I_ values were 0.51 kJ/mol during the heating and cooling processes, respectively.

**Figure 3:**
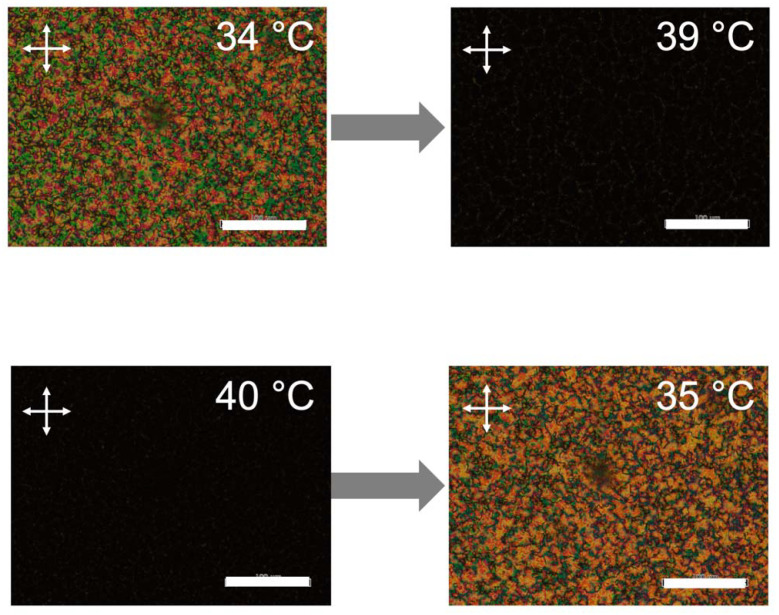
Polarized optical microscope (POM) images of the mixture of PMA(4OPB)-grafted ZnO nanorods and 5CB under crossed polarizers at the heating process (**top**) and cooling process (**bottom**). Scale bars, 100 µm. White crossed arrows show the direction of the polarizers.

In the case of their mixture (Figure 2c), the peaks due to the nematic to isotropic phase transition of PMA(4OPB)-grafted nanorods at 98 °C (heating) and 97 °C (cooling) disappeared. Instead, endothermic and exothermic peaks during the heating and cooling processes were observed at 35 °C, which was identical to the nematic to isotropic phase transition of 5CB. The melting point during the heating process was observed at 17 °C, but no crystallization peak was observed due to supercooling. Polarized optical microscope (POM) images of the mixture under crossed polarizers exhibited schlieren textures, which is characteristic of a nematic phase, at a temperature below 35 °C and became dark at higher temperatures (Figure 3). According to the DSC and POM measurements, the peaks of the DSC thermogram at 35 °C were attributable to the nematic to isotropic phase transition. The Δ*H*_N-I_ values of the mixture were 0.33 and 0.39 kJ/mol in the heating and cooling processes, respectively. The decrease in the phase transition enthalpies compared to 5CB suggested that the added PMA(4OPB)-grafted nanorods locally disordered the molecular orientation. Nevertheless, the single phase transition by DSC and the optically anisotropic textures, without significant segregation by POM, confirmed that PMA(4OPB)-grafted nanorods are miscible with a 5CB host and form a uniform nematic phase.

### 2.2. Homogeneous Alignment of ZnO Nanorods in Host LC

**Figure 4:**
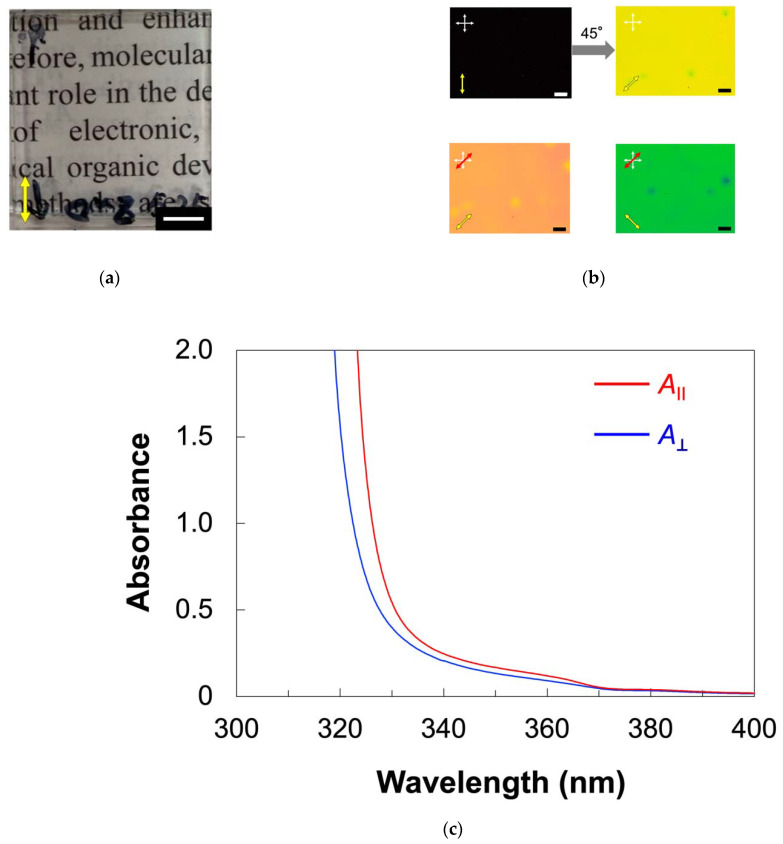
Optical properties of 5CB doped with PMA(4OPB)-grafted ZnO nanorods with a weight ratio of 20:1 in a glass cell with a homogeneous alignment layer. (**a**) Photograph. Scale bar, 5 mm. (**b**) POM images under crossed polarizers without (top) and with (bottom) a tint plate with a retardation of 137 nm. White crossed arrows show the direction of the polarizers. Yellow arrows show the rubbing direction. Red arrows show the direction of the tint plate. Scale bars, 200 µm. (**c**) Polarized UV–vis absorption spectra. $A_{∥}$ and $A_{⊥}$ are absorbances parallel and perpendicular to the rubbing direction.

**Figure A1:**
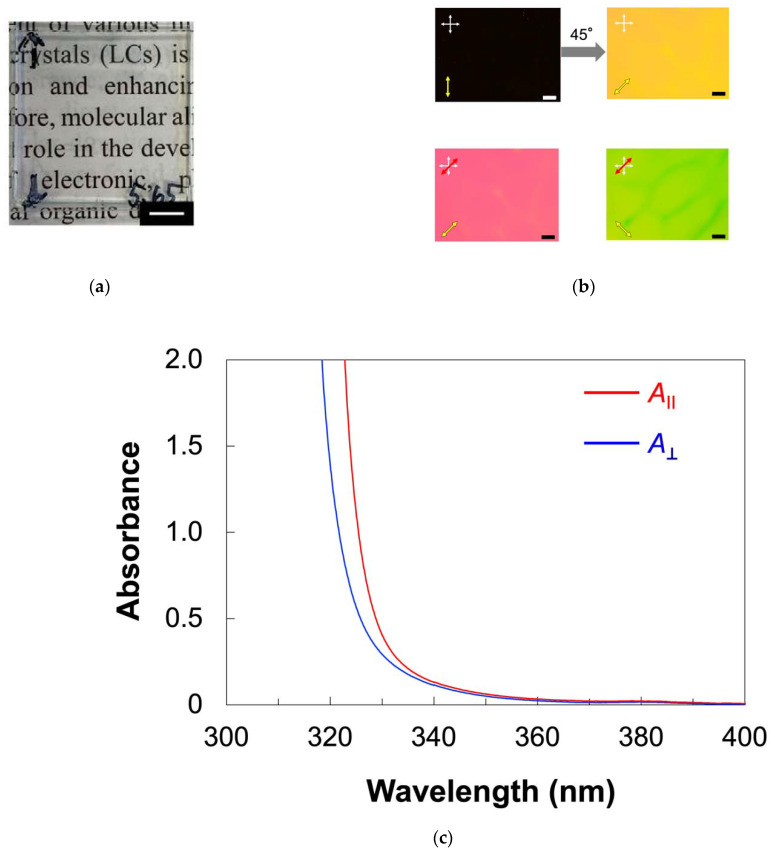
Optical properties of 5CB in a glass cell with a homogeneous alignment layer. (**a**) Photograph. Scale bar, 5 mm. (**b**) POM images under crossed polarizers without (top) and with (bottom) a tint plate with a retardation of 137 nm. White crossed arrows show the direction of the polarizers. Yellow arrows show the rubbing direction. Red arrows show the direction of the tint plate. Scale bars, 200 µm. (**c**) Polarized UV–vis absorption spectra. $A_{∥}$ and $A_{⊥}$ are the absorbances parallel and perpendicular to the rubbing direction.

The alignment behavior of PMA(4OPB)-grafted ZnO nanorods dispersed with a 5CB host was firstly investigated in an approximately 5 μm-thick cell coated with a homogeneous alignment layer which had been obtained by a rubbing treatment. Figure 4 shows the optical properties of the sample cell filled with the mixture. Similarly, in the 5CB cell (Figure A1a), the obtained cell was optically transparent, as shown in Figure 4a. The POM observation of the cell under crossed polarizers showed a clear contrast for every 45° rotation and the image became completely dark when the polarization direction was parallel or perpendicular to the rubbing direction, as indicated in the top images of Figure 4b. The molecular alignment direction was confirmed by the POM observation with a tint plate (retardation (*R*) = 137 nm). As shown in the bottom images of Figure 4b, the additive and subtractive color effects were found when the optical axis of the tint plate was parallel and perpendicular to the rubbing direction, respectively. The POM results indicated that the mesogens of the host LC were aligned parallel to the rubbing direction. The value of *R* for the mixture, measured using a Berek compensator, was 840 nm. Using the relation of *R* = *dΔn*, where d is the cell thickness (*d* = 5 μm) and *Δn* is birefringence, the value of *Δn* was calculated to be 0.16. Similarly, *Δn* of 5CB was determined to be 0.15 (Figure A1b). The almost identical *Δn* values, regardless of the existence of nanorods, suggested that PMA(4OPB)-grafted nanorods are well miscible with the host 5CB and form homogeneous alignment cooperatively without a significant disturbance of the molecular alignment.

To further investigate the alignment of ZnO nanorods as well as mesogens, we measured the polarized ultraviolet (UV)–visible (vis) absorption spectra (Figure 4c). We defined the absorbances parallel ($A_{∥}$) and perpendicular ($A_{⊥}$) to the rubbing direction, respectively. The absorption below 340 nm was derived from the cyanobiphenyl moieties of the host 5CB. The absorption band at around 350 nm, which was not observed for 5CB (Figure A1c), was assigned to the ZnO nanorods. The absorbance parallel to the rubbing direction was larger compared to the perpendicular direction for both bands. The order parameter (*S*), which showed the degree of the in-plane alignment, was calculated by using the following equation [35]:

(1) $$S=\frac{A_{∥}-A_{⊥}}{A_{∥}+2A_{⊥}}$$

The *S* value for ZnO nanorods was 0.09 at 355–360 nm. The *S* value for the host LC mesogens is not discussed here because the absorption of ZnO nanorods is also overlapped with that of mesogens. The results of POM and polarized UV–vis absorption spectra indicated that the ZnO nanorods were aligned cooperatively with the host LCs according to the rubbed alignment layer in micrometer-thick cells.

### 2.3. Homeotropic Alignment of ZnO Nanorods in the Host LC

**Figure 5:**
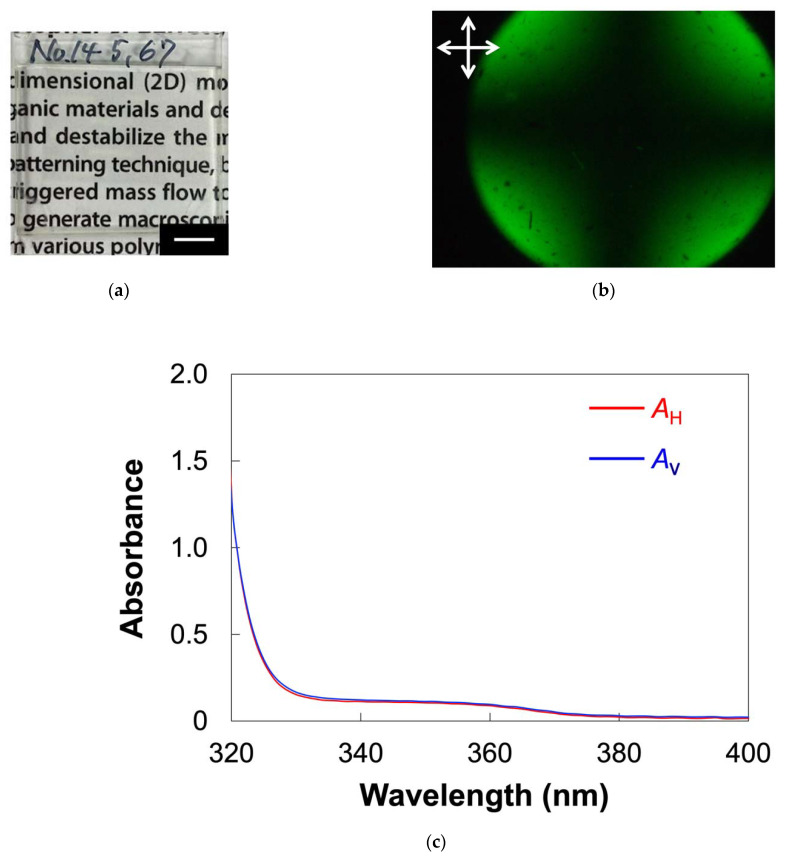
Optical properties of 5CB doped with PMA(4OPB)-grafted ZnO nanorods with a weight ratio of 20:1 in a glass cell with a homeotropic alignment layer. (**a**) Photograph. Scale bar, 5 mm. (**b**) Conoscopic POM image. White crossed arrows show the direction of the polarizers. Scale bar, 200 µm. (**c**) Polarized UV–vis absorption spectra. *A*_H_ and *A*_V_ are the absorbances parallel and perpendicular to the injection direction of the sample.

The ZnO nanorod-dispersed LC in a glass cell with a homeotropic alignment layer showed different optical properties from that in the homogeneously aligned cell, as shown in Figure 5. The glass cell was optically transparent, as shown in Figure 5a. A conoscopic POM image exhibited a clear isogyre (Figure 5b). The result indicated that host 5CB molecules in the cell have a homeotropic alignment. In addition, the alignment directions of both the host LC and PMA(4OPB)-grafted ZnO nanorods were evaluated by polarized UV–vis absorption spectroscopy. We determined the absorbances parallel (*A*_H_) and perpendicular (*A*_V_) to the direction of the sample injection, respectively. As shown in Figure 5c, the absorbance derived from ZnO and mesogens was identical regardless of the polarization direction. These results show that PMA(4OPB)-grafted ZnO nanorods are homeotropically aligned with host LCs in a micrometer-thick cell.

## 3. Materials and Methods

### 3.1. Materials

**Figure 6:**
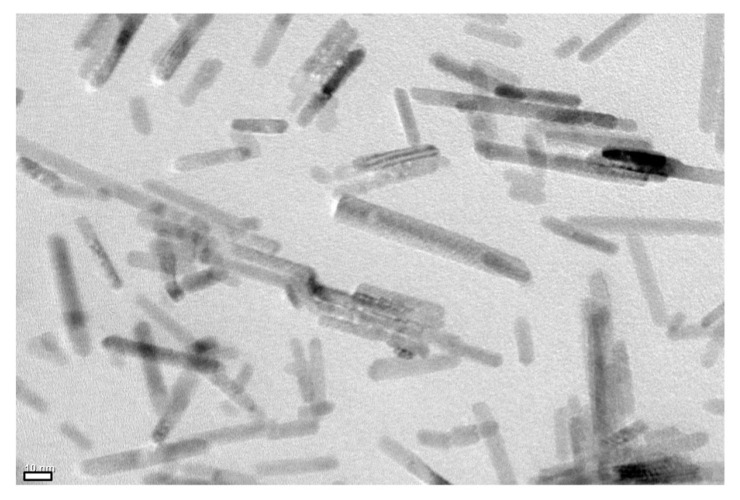
TEM image of the synthesized PMA(4OPB)-grafted ZnO nanorods. Scale bar, 10 nm.

A host LC (5CB) was provided by Merck, Darmstadt, Germany, and used without further purification. ZnO nanorods grafted with a nematic LC polymer PMA(4OPB) were synthesized as previously reported [34]. ZnO nanorods with an average diameter of 7 nm and a length of 50 nm were modified with initiator moieties for atom transfer radical polymerization (ATRP). The surface density of the ATRP initiator moieties was controlled to be 0.79 nm^−2^, which was determined by X-ray fluorescence (XRF) analysis. A nematic LC polymer PMA(4OPB) was grafted from the initiator-modified ZnO nanorods by ATRP with a feed molar ratio of 4-[4-(4-methoxyphenyloxycarbonyl)phenoxy]butyl methacrylate to the initiator moieties equal to 100. The monomer conversion measured by ^1^H NMR was 33%. The resultant PMA(4OPB)-grafted ZnO nanorods were observed using a transmission electron microscope (TEM), as shown in Figure 6. The precursor solution of the homogeneous alignment layer (AL1254) was supplied by JSR Corporation, Tokyo, Japan, and that for the homeotropic alignment layer (Sunever) was supplied by Nissan Chemical Corporation, Tokyo, Japan.

### 3.2. Sample Preparation

**Figure 7:**
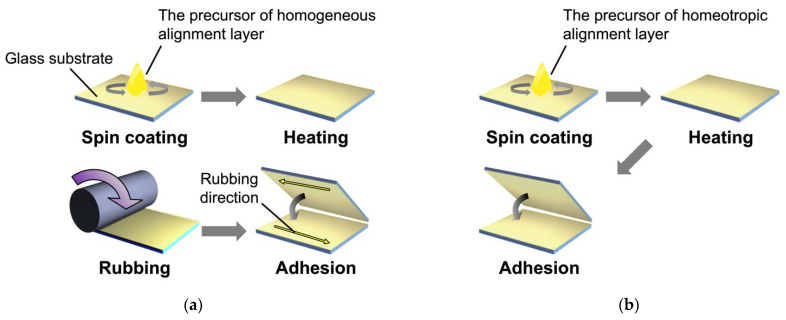
Schematic illustrations of the preparation of glass cells with homogeneous (**a**) and homeotropic (**b**) alignment layers.

**Figure 8:**
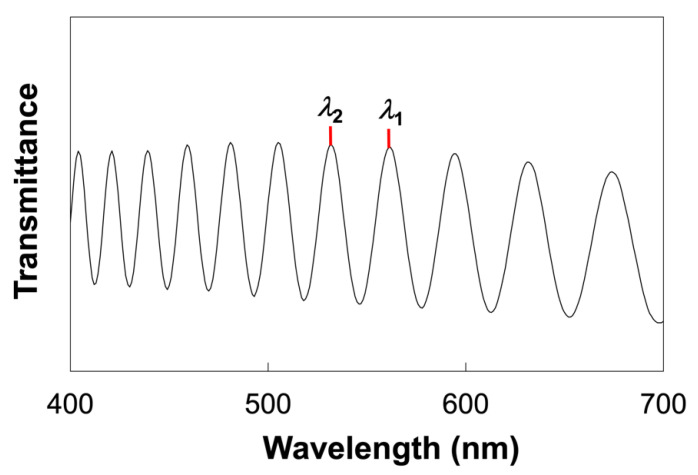
Typical transmission spectrum of the fabricated cell. *λ***_1_** and *λ***_2_**: peak wavelength.

Glass cells with homogeneous and homeotropic alignment layers were fabricated according to the procedure shown in Figure 7. Glass substrates (25 mm × 15 mm) were ultrasonically cleaned with 2-propanol for 30 min and treated with a UV-ozone cleaner (NL-UV42, Nippon Laser & Electronics Lab Co. Ltd., Nagoya, Japan) for 10 min. The precursor solutions for the alignment layers were spincoated on the cleaned glass substrates and heated at 220 °C for 1 h. The glass substrates with the homogenous alignment layer were rubbed by a rubbing machine (MRG-100, EHC Co., Ltd., Hachioji, Japan). Glass cells were fabricated by adhering a pair of alignment layer-coated glass substrates with glue containing 5 μm-thick silica spacers (Thermo Scientific, 9000 Series, #9005, Thermo Fisher Scientific, Waltham, MA, USA). The actual thickness of the prepared glass cells was determined by UV–vis spectroscopy based on the Fabry–Perot method. A typical transmission spectrum is shown in Figure 8. The thickness (*d*) was calculated using the wavelengths of the interference maximum (*λ*_1_, *λ*_2_) by the following equation [36]:

(2) $$d=\frac{\lambda_{1}\lambda_{2}}{2\left(\lambda_{1}-\lambda_{2}\right)}$$

**Figure 9:**
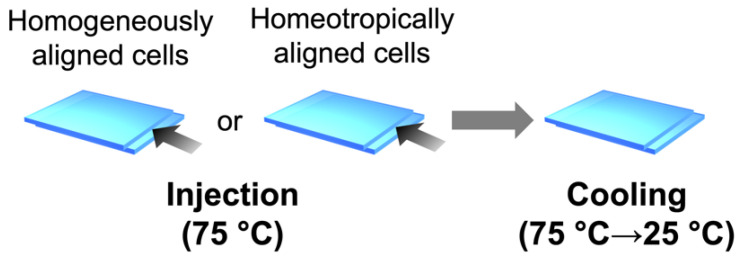
Schematic illustration of the injection of LCs into the glass cells.

The mixture of PMA(4OPB)-grafted ZnO nanorods and 5CB with a weight ratio of 1:20 was dispersed in tetrahydrofuran (THF) and stirred for 1 h at room temperature. After the removal of THF under vacuum for 6 h, the mixture was further treated with an ultrasonicator (VS-02RD, Velvo-Clear, Tokyo, Japan) for 30 min at room temperature to improve the dispersity of PMA(4OPB)-grafted ZnO nanorods in the host 5CB. The sample mixture was injected into the hand-made glass cells by capillary action at 75 °C and cooled down to 25 °C at a rate of 10 °C/min, as shown in Figure 9.

### 3.3. Characterization Equipment

Differential scanning calorimetry (DSC) was performed using an Exstar DSC7000X differential scanning calorimeter (Hitachi High-Tech Corp., Tokyo, Japan). Polarized optical microscope (POM) images were obtained by a BX 50 polarized optical microscope (Olympus Corp., Tokyo, Japan) equipped with a hot stage (HS82, Mettler Toledo, Greifensee, Switzerland) and a tint plate (U-TP137, Olympus Corp., Tokyo, Japan) or a Berek compensator (U-CBE, Olympus Corp., Tokyo, Japan). Polarized UV–vis absorption spectra were measured by a UV–vis absorption spectrophotometer (V-670, JASCO Corp., Hachioji, Japan). XRF analysis was performed using an X-ray fluorescence spectrometer (ZSX Primus II, Rigaku Corp., Akishima, Japan). ^1^H NMR spectra were recorded by an NMR spectrometer (Avance III, 400 MHz, Bruker Biospin, Bruker, Billerica, MA, USA). Transmission electron microscopy was performed with a JEM-2100 microscope (JEOL Ltd., Akishima, Japan).

## 4. Conclusions

In this study, we investigated the alignment control of nematic LC polymer-grafted nanorods dispersed in host LCs in micrometer-thick cells. The surface-grafted nanorods were well miscible with the host LCs and formed a uniform LC phase, as confirmed by DSC analysis. POM and polarized UV–visible absorption spectroscopy revealed the homogeneous and homeotropic alignment of the surface-grafted nanorods parallel to the host LCs in 5 μm-thick cells treated with alignment layers. Furthermore, the birefringence of the host LCs dispersed with surface-grafted nanorods was almost identical to that of 5CB, which suggests a cooperative interaction of grafted nematic LC polymers with host LCs without significant segregation. The results reported herein will contribute to the development of various microscale devices by enabling us to effectively utilize the anisotropic properties of well-aligned nanomaterials.

## Data Availability

The authors confirm that the data supporting the findings of this study are available within the article.

## Sample Availability

Samples of the compounds are not available from the authors.

## Appendix A

A homogeneous alignment cell of 5CB was prepared for comparison. The optical properties are shown in Figure A1.
